# Carnosic Acid Induces Antiproliferation and Anti-Metastatic Property of Esophageal Cancer Cells via MAPK Signaling Pathways

**DOI:** 10.1155/2021/4451533

**Published:** 2021-11-16

**Authors:** Sicong Jiang, Yinda Qiu, Zhaozhen Wang, Yulong Ji, Xiaofang Zhang, Xiaosong Yan, Zhiqiang Zhan

**Affiliations:** ^1^Division of Thoracic and Endocrine Surgery, University Hospitals and University of Geneva, Geneva 41211, Switzerland; ^2^School of Pharmaceutical Sciences, Wenzhou Medical University, Wenzhou, Zhejiang, China; ^3^Department of Clinical Medicine, Jiangxi Health Vocational College of China, Nanchang, Jiangxi, China; ^4^Jiangxi Key Laboratory of Translational Cancer Research, Jiangxi Cancer Hospital of Nanchang University, Nanchang, Jiangxi, China; ^5^Department of Pathology, Jiangxi Cancer Hospital of Nanchang University, Nanchang, Jiangxi, China; ^6^Department of Thoracic Surgery, Jiangxi Cancer Hospital of Nanchang University, Nanchang, Jiangxi, China; ^7^Department of Oncology, Jiangxi Pingxiang People's Hospital, Pingxiang, Jiangxi, China

## Abstract

**Background:**

Carnosic acid (CA) is a polyphenolic diterpene extracted from rosemary. Reports have shown that CA possesses anticancer activity. However, whether CA inhibits esophageal squamous cell carcinoma, an aggressive type of esophageal cancer, remains untested.

**Methods:**

The effects of CA on cell survival, migration, and apoptosis were evaluated by a combination of MTT, colony formation assay, flow cytometry, and Transwell assay. The potential signaling pathways involved were investigated via Western blot assay.

**Results:**

CA dose-dependently inhibited cell proliferation, apoptosis, migration, and colony formation. Mechanistically, CA arrested the cell cycle at G2/M phase, promoted cell apoptosis, induced DNA damage, and inhibited the MAPK signaling pathways.

**Conclusion:**

Our results suggest that CA is a potential anticancer drug for esophageal squamous cell carcinoma.

## 1. Introduction

Esophageal cancer is one of the most common malignant tumors in the digestive system. There are two main pathological types of esophageal cancer: esophageal adenocarcinoma (EAC) and esophageal squamous cell carcinoma (ESCC) [1–4]. The early vague and nonspecific symptoms of ESCC have contributed to its high morbidity and mortality [5]. Despite continuous improvement in treatment regimens, including surgery, chemotherapy, and radiotherapy, the 5-year survival rate remains fluctuating between 15% and 25% [6–8]. In addition, chemotherapeutic agents often have unbearable side effects. Thus, there is an urgent need to develop natural and safer compounds for the treatment of ESCC.

Carnosic acid (CA), a polyphenolic diterpene derived from the rosemary plant, possesses anti-inflammatory, antiviral, antioxidative, and antitumor activities [9]. Reports have shown that CA inhibited proliferation and migration of breast cancer and melanoma cells, arrested the cell cycle at the G2/M phase through suppression of cyclin A expression in leukemia and intestinal cancer, and induced apoptosis in prostate cancer and tumors of the human central nervous system [10, 11]. This antitumor effect may be mediated through inhibition of the MAPK signaling pathways. This pathway represents a large family of serine/threonine kinases that, upon the reception of a stimuli, trigger a cascade of phosphorylation leading to specific cellular responses [12] and also plays a critical role in tumor progression and metastasis by induction of proteolytic enzymes that degrade the ECM (a key marker of invasion carcinoma), enhancement of cell migration, initiation of several prosurvival genes, and maintenance of tumor growth [13]. In addition, combination of CA with other drugs, such as curcumin, adriamycin, and carmustine, increased the antitumor effects of the latter compounds [14]. In this report, we studied the potential use of CA in the treatment of ESCC and explored the molecular mechanisms underlying its tumor-suppressive effect.

## 2. Materials and Methods

### 2.1. Cell Culture

Human esophageal squamous carcinoma cell line, KYSE-150, and normal human liver cells, MIHA, were obtained from the Institute of Biochemistry and Cell Biology, Chinese Academy of Sciences (Shanghai, China). KYSE-150 cells were cultured in RPMI-1640 (Gibco, Eggenstein, Germany) supplemented with 10% fetal bovine serum (FBS) (Gibco, Eggenstein, Germany) and 1 × penicillin/streptomycin (Thermo Fisher Scientific, Carlsbad, CA, USA). MIHA cells were grown in Dulbecco's modified Eagle's medium (DMEM) (Gibco, Eggenstein, Germany) supplemented with 10% FBS and 1 × penicillin/streptomycin. All cells were cultured at 37°C in a 5% CO_2_ atmosphere.

### 2.2. Cell Viability Assay

KYSE-150 (6500 cells/well) and MIHA (6000 cells/well) were seeded in 96-well plates. Cells were incubated overnight and treated with increasing concentrations of CA (10, 20, and 40 *μ*M) for 48 h. The cell viability was determined using 3-(4,5-di-methylthiazol-2-yl)-2,5-diphenyl-2 tetrazolium bromide (MTT) assay. In brief, cells were incubated with MTT reagent at 37°C for 4 h, and 100 *μ*l DMSO was added to each well to dissolve formazan crystals. The absorbance was read at 490 nm on a spectrophotometer (DTX880, Beckman Coulter, CA, USA).

### 2.3. Colony Formation Assay

KYSE-150 cells were seeded at a density of 1000 cells/well in 12-well plates and allowed to attach overnight. Cells were treated with different concentrations of CA (10, 20, and 40 *μ*M) for 15 days at 37°C. The cells were washed with PBS, fixed with 4% paraformaldehyde for 15 min, and stained with crystal violet 10 min at room temperature. Colonies were counted using a stereomicroscope.

### 2.4. EdU Staining Assay

KYSE-150 cells were seeded in 6-well plates the day before the experiment. Cells were treated with 10, 20, and 40 *μ*M CA for 48 h. The EdU solution was added (final concentration of 10 *μ*M), and cells were continued to incubate for 2 h. Then, cells were processed for EdU staining using the EdU Proliferation kit (Beyotime, China) following the vendor's instructions. Cells were then observed under fluorescence microscopy (Nikon).

### 2.5. Cell Cycle Analysis

KYSE-150 cells (2.5 × 10^5^) were seeded in 6-well plates and incubated with 10, 20, and 40 *μ*M CA for 48 h. Cells were fixed in 70% ethanol for 24 h, washed with cold PBS, and incubated with 50 *μ*g/mL PI (propidium iodide) and 100 *μ*g/mL RNase solution in PBS for 30 min at room temperature. Cells were analyzed on a flow cytometer (BD FACSalibur, BD Biosciences).

### 2.6. Cell Apoptosis Analysis

Apoptosis was analyzed using the apoptosis detection kit (BD Biosciences, USA) following the vendor's instructions. KYSE-150 cells were treated with CA (10, 20, and 40 *μ*M) for 48 h and washed with PBS once and Annexin V-binding buffer once. The cells were simultaneously incubated with fluorescein-labeled Annexin V and PI for 15 min at room temperature. Cells were then washed once again with Annexin V-binding buffer and resuspended with the same buffer, which were analyzed on a FACSCalibur (BD Biosciences, MD, USA).

### 2.7. Transwell Assay

Cells were treated with CA at 10, 20, and 40 *μ*M for 48 h, which were then digested, resuspended, and diluted with serum-free medium to a concentration of 1 × 10^5^/100 *μ*l. Subsequently, 600 *μ*l of RPMI-1640 containing 10% FBS was added into the lower chamber and cultured in a 37°C incubator. Cells on the top of the membrane were removed using Q-tips, and cells on the bottom of the membrane were fixed with 4% paraformaldehyde for 15 min at room temperature and washed with double distilled (DD) water. The cells were stained with crystal violet for 5 min and rinsed with DD water and 30% glacial acetic acid to dissolve crystal violet. The plate was read at 560 nm. Cell migration rate was calculated as follows: average OD of treated cells/average OD of control unit) × 100%.

### 2.8. Immunofluorescence (IF) Assay

Cells on coverslips were fixed with 4% paraformaldehyde, permeabilized in 0.5% Triton X-100 (in 1 × PBS), and incubated overnight at 4°C with primary antibody against 53BP1 (CST, Danvers, MA). Cells were washed three times and incubated with DyLight 549-conjugated anti-rabbit secondary antibodies for 1 h at room temperature. Cells were stained with DAPI, and images were acquired using Nikon Ti microscopy.

### 2.9. Western Blot Assay

Cells were grown on 6-well plates and were treated with 10, 20, and 40 *μ*M CA for 48 h. The cells were then washed with PBS and lysed using cell lysis buffer. The cell lysates were quantitated using the BCA method (BioRad, CA, USA). An equal amount of cell lysates was resolved on SDS-PAGE and electroblotted onto polyvinylidene difluoride membranes. The membranes were blocked using 5% nonfat milk at room temperature for 2 h and incubated with primary antibodies at 4°C overnight. The membranes were incubated with the peroxidase-conjugated secondary antibodies for 1.5 h at room temperature. The immunoreactive bands were visualized using an ECL detection kit (BioRad Laboratories, CA, USA).

### 2.10. Statistical Analysis

All data were analyzed by GraphPad Prism 7.0. The means of multiple groups were compared by one-way analysis of variance, and the means of two groups were compared with the independent samples *t*-test. Values are expressed as the mean ± SD of three independent experiments, with ^*∗*^*p* < 0.05,  ^*∗∗*^*p* < 0.01,  and  ^*∗∗∗*^*p* < 0.001.

## 3. Results

### 3.1. Effects of CA Treatment on Cell Survival of KYSE-150 Cells

**Figure 1:**
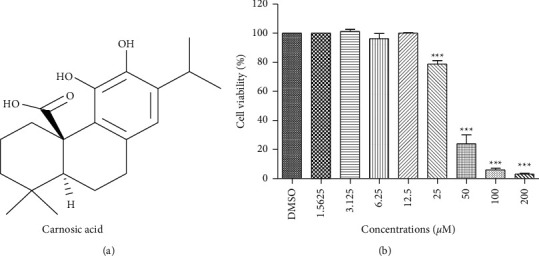
Evaluation of cytotoxicity and potency of CA. (a) The chemical structure of carnosic acid. (b) KYSE-150 viability was assessed using the MTT assay 48 h after treatment. Data were the means ± SD of three independent experiments (^*∗∗∗*^*p* < 0.001 vs control group).

CA is a natural benzenediol abietane diterpene found in rosemary, with its chemical structure as shown in Figure 1(a). To evaluate the effect of CA on cell survival, KYSE-150 cells were treated with increasing concentrations of CA as indicated in Figure 1(b) for 48 h and cell viability was analyzed by MTT assay. CA at <25 *μ*M had no significant effect on cell viability, which was decreased to 80% at 25 *μ*M, and CA at >25 *μ*M dose-dependently decreased cell viability. To evaluate the potency of CA, the half-maximal inhibitory concentration (IC50) was compared with that of the two most commonly used chemotherapeutic drugs, 5-fluorouracil (5-FU) and cisplatin (DDP). As shown in Table 1, the IC50 of CA for KYSE-150 cells was 29.87 ± 4.38 *μ*M, compared to 65.98 ± 1.39 of 5-FU and 79.21 ± 2.02 of DDP and IC50 for MIHA cells was >200 *μ*M, compared to >200 of 5-FU and 16.64 ± 0.39 of DDP. These results suggest that CA at less than 25 *μ*M had no obvious cytotoxicity and is a potent anticancer drug in KYSE-150 cells.

### 3.2. CA Inhibits the Proliferation of KYSE-150 Cells

**Figure 2:**
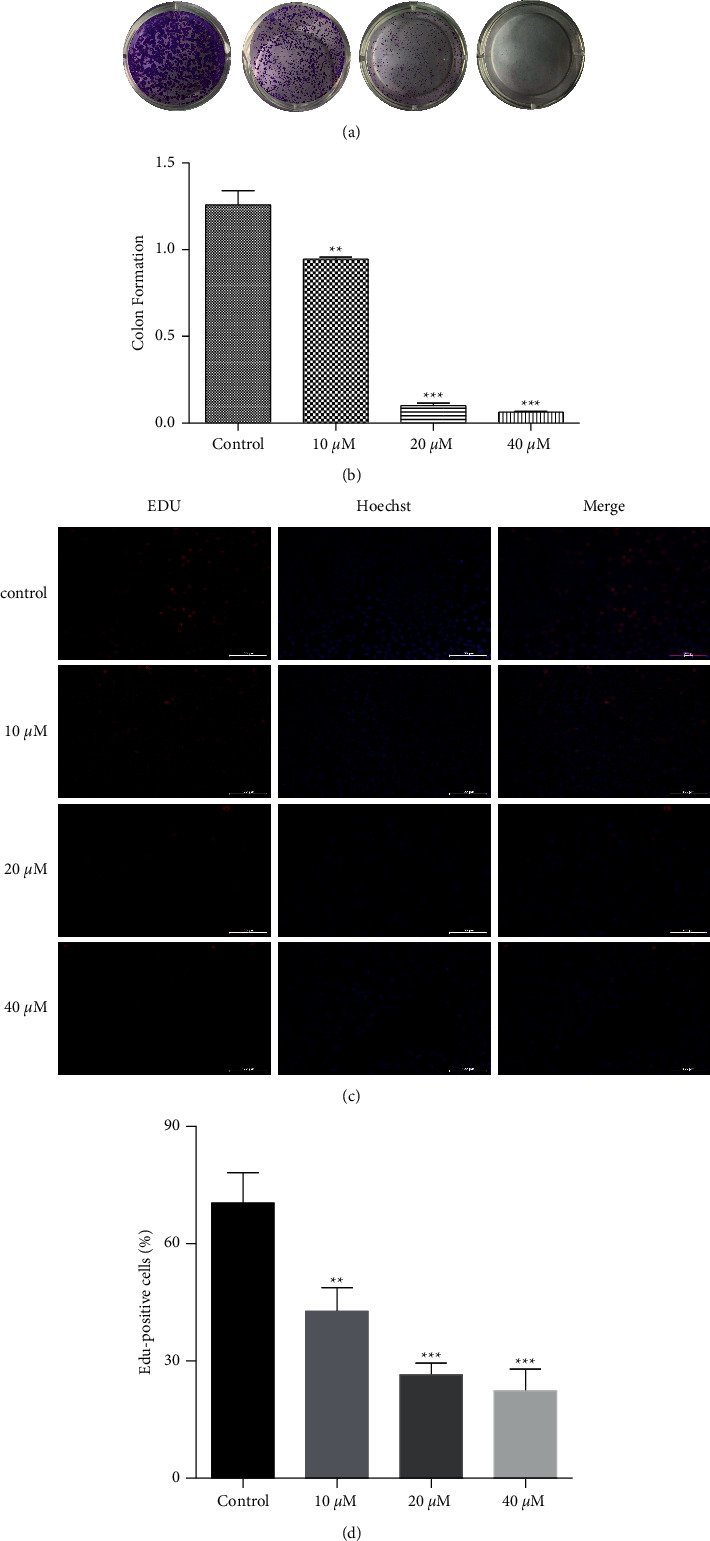
CA inhibits proliferation of KYSE-150 cells. (a) Cells were treated with CA and grown for 15 days. CA dose-dependently decreased colony formation. (b) Quantification of colony formation in (a). (c) Representative images showing EdU-positive cells after CA treatment for 48 h. (d) Quantification of (c). The statistical significance was calculated using the unpaired student's two-tailed *t*-test with the *p* values (^*∗∗*^*p* < 0.01 and  ^*∗∗∗*^*p* < 0.001 vs control group).

To evaluate the effect of CA on colony formation capability, the colony formation assay was performed using KYSE-150 cells as described in the Materials and Methods section. As shown in Figures 2(a) and 2(b), CA inhibited colony formation in a dose-dependent manner. To evaluate the effect of CA on cell proliferation, KYSE-150 cells were treated with EdU as described in the Materials and Methods section. As shown in Figures 2(c) and 2(d), CA dose-dependently inhibited cell proliferation.

### 3.3. CA Arrests Cell Cycle at the G2/M Phase

**Figure 3:**
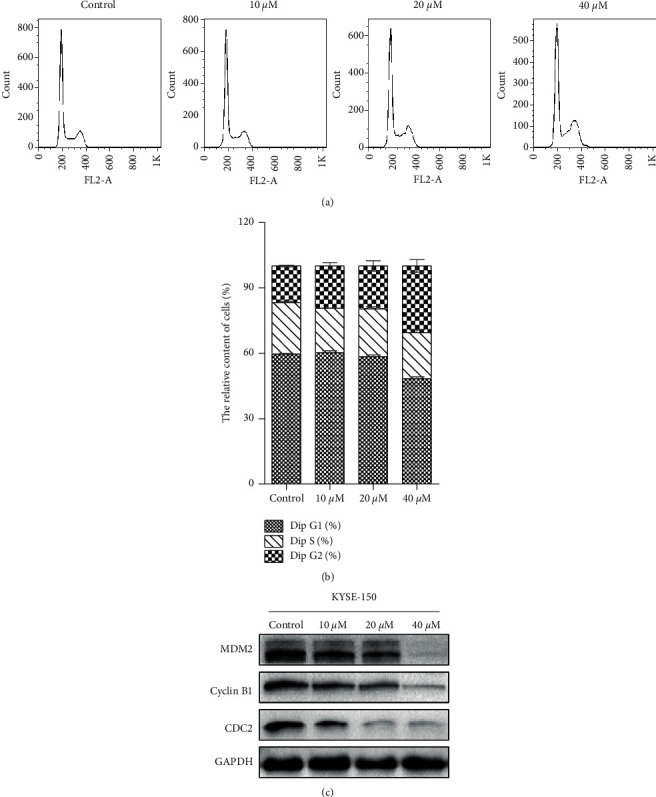
CA arrests KYSE-150 cells at the G2/M phase. KYSE-150 cells were treated with different concentrations of CA for 48 h. (a) The cell cycle was measured using flow cytometry. (b) Quantification of flow cytometry data in (a). (c) The expressions of MDM2, cyclin B1, and CDC2 were measured using Western blot. Values were mean ± SD of at least three independent experiments (^*∗*^*p* < 0.05 and  ^*∗∗*^*p* < 0.01 vs control group).

To further explore the molecular mechanisms of CA-induced inhibition of cell proliferation, KYSE-150 cells were treated with 10, 20, and 40 *μ*M of CA for 48 h and the cell cycle was analyzed by flow cytometry. As shown in Figures 3(a) and 3(b), CA arrested KYSE-150 cells at the G2/M phase in a dose-dependent manner. It is worth noting that CA treatment increased the percentage of cells at the G2/M phase and decreased the percentage of cells at the G0/G1 phase relative to controls. Cells were then treated with 5, 10, and 20 *μ*M of CA, and G2/M phase-related proteins were evaluated by Western blot. CA dose-dependently decreased the expression of cyclin B1, MDM2, and CDC2 (Figure 3(c)). Taken together, CA-induced inhibition of cell proliferation occurs most likely through suppression of G2/M phase-related proteins, leading to cell cycle arrest at the G2/M phase.

### 3.4. CA Provokes Strong DNA Damage Response

**Figure 4:**
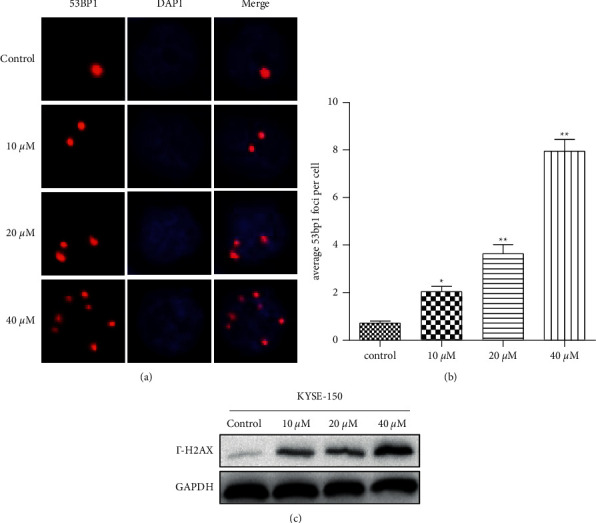
CA provokes intense DNA damage response in KYSE-150 cells. (a) Representative images showing CA treatment provokes intense DNA damage response by immunofluorescence. (b) Quantification of (a) showing that CA dose-dependently increased the DNA damage (more than 200 cells were examined in each group). (c) Western blot showing that CA dose-dependently increased the expression of *γ*-H2AX. Values were presented as the mean ± SD of at least three independent experiments (^*∗*^*p* < 0.05 and  ^*∗∗*^*p* < 0.01 vs control group).

DNA damage induced by anticancer drugs can block cells at the G2/M phase, which prevents cells from entering into mitosis, leading to their anticancer effects. To investigate whether CA-induced G2/M phase arrest was caused by DNA damage, KYSE-150 cells were treated with CA for 48 h and P53 binding protein 1 (53BP1), a widely used marker for DNA double-strand breaks, was evaluated by immunofluorescence (IF) assay. As shown in Figure 4, CA dose-dependently increased the number of 53BP1-positive foci, with CA at 40 *µ*M reaching ∼ eight 53BP1-positive foci per nucleus. When phosphorylation of the Ser-139 residue of the histone variant H2AX (*γ*-H2AX) occurs, a molecular marker of DNA double-strand break was evaluated by Western blot. CA dose-dependently increased the expression of *γ*-H2AX. The results suggested that CA provokes a strong DNA damage repair response in KYSE-150 cells.

### 3.5. CA Induces Apoptosis of KYSE-150 Cells

**Figure 5:**
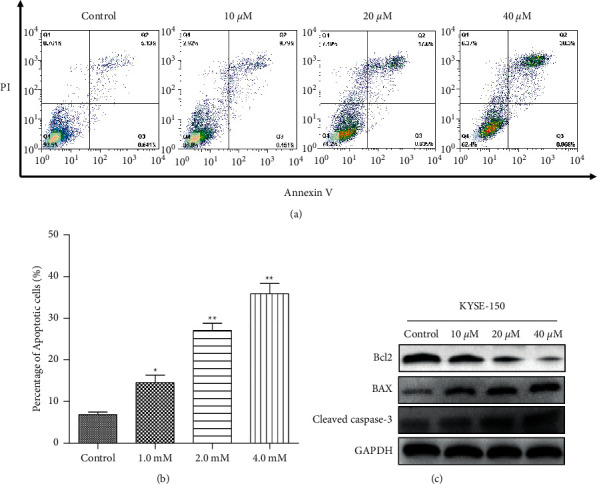
CA promotes apoptosis of KYSE-150 cells. (a) The apoptosis of KYSE-150 was analyzed by flow cytometry. (b) Quantification of flow cytometry data in (a). (c) The expression of Bcl2 was inhibited, while that of Bax and cleaved caspase-3 was activated in a dose-dependent manner by Western blot. GAPDH was used as loading control. Values are average ± SD of three independent experiments (^*∗*^*p* < 0.05 and  ^*∗∗*^*p* < 0.01 vs control group).

Severe DNA damage usually leads to cell cycle arrest and apoptosis. To test this, KYSE-150 cells were treated with CA as indicated in Figure 5, and flow cytometry assay was performed to analyze the cell apoptosis. As expected, CA-induced KYSE-150 cell apoptosis in a dose-dependent manner (Figure 5(a)). When apoptosis-related proteins, Bax, Bcl2, and cleaved caspase-3 were analyzed by Western blot, CA dose-dependently decreased the expression of Bcl2 and simultaneously increased the expression of both Bax and cleaved caspase-3, respectively (Figure 5(c)). These results indicated that CA induces cell apoptosis by regulating the expression of apoptosis-related proteins.

### 3.6. CA Inhibits Metastasis and Invasion of KYSE-150 Cells via Suppressed MAPK Signaling Pathway

**Figure 6:**
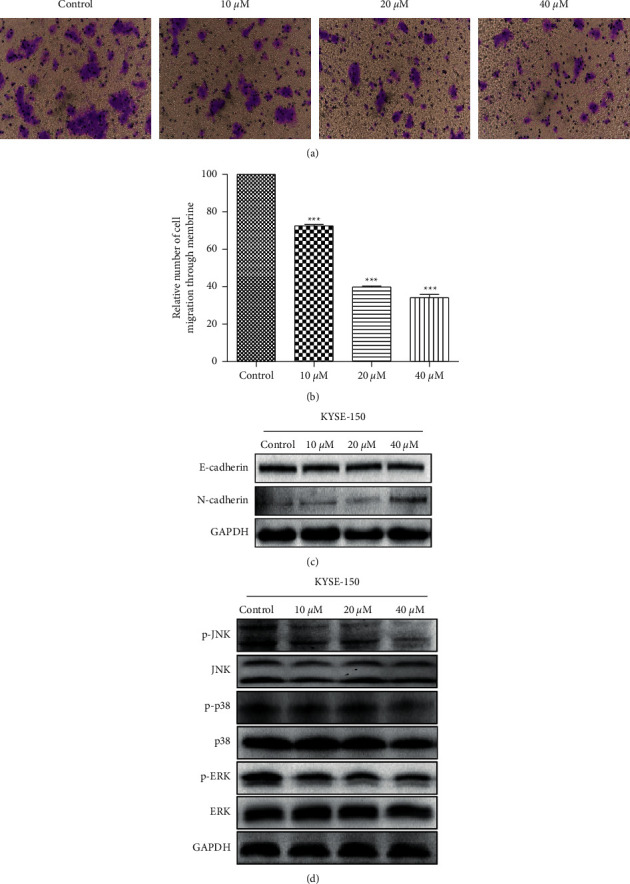
CA inhibits the migration and invasion of KYSE-150 cells via suppressing the MAPK signaling pathway. (a) CA dose-dependently inhibits cell migration as evaluated by Transwell assay. (b) Quantification of cell migration in the Transwell assay in (a). (c) CA dose-dependently decreases the expression E-cadherin and simultaneously increases the expression of N-cadherin, respectively, by Western blot. (d) CA dose-dependently decreases the levels of p-JNK, p-ERK, and p-38 but does not affect those of total JNK, p-38, and ERK by Western blot.

Cell migration and invasion play an important role in cancer metastasis. To evaluate the effects of CA on cell migration and invasion, KYSE-150 cells were treated with CA at 10, 20, and 40 *μ*M for 24 h and Transwell assay was performed. As shown in Figures 6(a) and 6(b), CA dose-dependently decreased cell migration. Recent studies have shown that inappropriate activation of the epithelial-to-mesenchymal transition (EMT) is associated with increased tumor invasion and metastasis [15, 16]. To explore whether CA inhibits EMT, KYSE-150 cells were treated with CA and Western blot assay was used to determine the expression of the key proteins involved in the EMT process. However, as shown in Figure 6(c), CA did not inhibit tumor cell metastasis and invasion via an EMT pathway. To investigate the potential singling pathways involved in CA-induced inhibition of cell proliferation and migration, components of the signaling pathways were analyzed by Western blot. As shown in Figure 6(d), CA dose-dependently decreased the expression of p-ERK, p-JNK, and p-38 but did not affect that of total JNK, p-38, and ERK. These results suggest that CA inhibits cell metastasis and invasion through suppression of ERK, JNK, and p-38 signaling pathways.

## 4. Discussion

ESCC is one of the most aggressive cancers and ranked the sixth leading cause of cancer death in the world [17]. Although progress has been made in clarifying the molecular mechanisms of its pathogenesis, ESCC still carries high mortality rates, early metastasis, and poor prognosis [18, 19]. CA is a functional ingredient of rosemary that has been used as an antioxidant food for many years [20, 21]. Recently, research has shown that CA exhibits antitumor activity in colon cancer, breast cancer, and skin tumors through inhibition of cell proliferation, invasion, and metastasis and induction of apoptosis and ROS production [22]. In the present study, we demonstrated that CA suppressed the malignant phenotypes of ESCC cells. Mechanistically, the tumor-suppressive effect of CA was mediated mainly by cell cycle arrest at the G2/M phase, promotion of severe DNA damage and apoptosis, and activation of MAPK multiple signaling pathways.

As a naturally occurring anticancer compound, CA has been studied for its cytotoxic effect in normal cells and its anticancer activity in different tumors. CA was demonstrated to decrease cell viability in a dose-dependent manner, with an IC50 of 25.6 to 96 *µ*M in breast cancer MCF7 cells and 19.6 *µ*M and 22.9 *µ*M in prostate cancer cell lines LNCaP and 22Rv1 cells, respectively [23, 24], suggesting it is a very potent anticancer compound. In agreement with the previous studies, we found that CA at less than 25 *µ*M did not have obvious cytotoxicity, and concentrations larger than 25 *µ*M dose-dependently decreased cell viability of an ESCC cell line, KYSE-150 (Figure 1(b)).

CA has been shown to inhibit the malignant phenotypes of cancer cells in different cancers, and its molecular mechanisms seem to be cancer type-dependent. CA has been reported to inhibit cell proliferation through cell cycle arrest at the G0/G1 phase in melanoma cancer [25], at the G2 phase in human glioma [26], and at the G1 phase in estrogen receptor (ER)-negative human breast cancer cells [27]. This anticancer activity was mediated by the capabilities of CA to activate p21-mediated signaling pathway [25] and induced apoptosis and production of reactive oxygen species (ROS) [28], inhibited the EMT [29], enhanced the anticancer effects of other compounds [26], and sensitized TRAIL-mediated apoptosis [30]. Here, we showed that CA dose-dependently inhibited the malignant phenotypes, including cell proliferation, migration, and colony formation, in an ESCC cell line, KYSE-150 (Figure 2). In addition, CA dose-dependently decreased the expression of G2/M phase-related proteins, MDM2, cyclin B1, and CDC2 (Figure 3). Anticancer drug-induced DNA damage can activate p53-dependent pathways, blocking the cell cycle at the G2/M phase and inhibiting cell proliferation [31–33]. Consistently, we demonstrated that CA could induce severe DNA damage, showing a dose-dependent increase in P53BP1-positive foci per nucleus after CA treatment (Figure 4), which was corroborated by an increased expression of *γ*-H2AX, a molecular marker of DNA double-strand break. It is well-known that cells with irreversible DNA damage and cell cycle arrest will undergo apoptosis [34, 35]. In line with this, we observed that CA dose-dependently increased KYSE-50 cell apoptosis compared with a nontreated control group by flow cytometry analysis (Figures 5(a) and 5(b)). These data showed a dose-dependent decrease in the expression of apoptosis-inhibiting factor, Bcl2, and a simultaneous increase in the expression of apoptosis-promoting factors, Bax and cleaved caspase-3, by Western blot (Figure 5(c)). MAPK signaling pathways play an important role in the initiation of tumor cell invasion and metastasis [36]. Indeed, CA dose-dependently inhibited cell migration (Figures 6(a) and 6(b)), which was most likely mediated by the suppression of ERK, p-38, and JNK signaling pathways (Figure 6(d)).

## 5. Conclusion

CA dose-dependently inhibited the malignant phenotypes of an ESCC cell line, KYSE-150, which is most likely mediated by multiple molecular mechanisms, including cell cycle arrest, promotion of apoptosis, inhibition of cell migration, and a coordinated inhibition of ERK, p-38, and JNK signaling pathways. Our observations suggest that CA could be an efficacious drug for ESCC treatment; however, additional testing is warranted in large animal models and clinical trials.

## Figures and Tables

**Table 1:** *IC*50 (*μ*M) values were determined via the MTT assay.

Cell lines/compounds	IC50 (*μ*M)	
	MIHA	KYSE-150
Carnosic acid	>200	29.87 ± 4.38
5-Fluorouracil	>200	65.98 ± 1.39
DDP	16.64 ± 0.39	79.21 ± 2.02

IC50 (*μ*M) values were drug concentrations necessary for 50% inhibition of cell viability. Data are presented as “mean ± SD” from at least three independent experiments in triplicates. The drug treatment period was 48 h.

## Data Availability

The data that support the findings of this study will be made available upon request to the corresponding author.

## Abbreviations

CA: Carnosic acid
ESCC: Esophageal squamous cell carcinoma
EAC: Esophageal adenocarcinoma
EMT: Epithelial-to-mesenchymal transition
DD: Double distilled
IF: Immunofluorescence
CIS: Cisplatin
5-FU: 5-Fluorouracil.
